# The Impact of Acute Brain Dysfunction in the Outcomes of Mechanically Ventilated Cancer Patients

**DOI:** 10.1371/journal.pone.0085332

**Published:** 2014-01-22

**Authors:** Isabel C. T. Almeida, Márcio Soares, Fernando A. Bozza, Cassia Righy Shinotsuka, Renata Bujokas, Vicente Cés Souza-Dantas, E. Wesley Ely, Jorge I. F. Salluh

**Affiliations:** 1 Intensive Care Unit and Postgraduate Program, Instituto Nacional de Câncer, Rio de Janeiro, Rio de Janeiro, Brazil; 2 D'Or Institute for Research and Education, Rio de Janeiro, Rio de Janeiro, Brazil; 3 Intensive Care Lab, Instituto de Pesquisa Evandro Chagas, IPEC, Fundação Oswaldo Cruz, Rio de Janeiro, Rio de Janeiro, Brazil; 4 Vanderbilt University School of Medicine, Nashville, Tennessee, United States of America; 5 Veteran's Affairs Tennessee Valley Geriatric Research Education Clinical Center (VA-GRECC), Nashville, Tennessee, United States of America; Johns Hopkins Hospital, United States of America

## Abstract

**Introduction:**

Delirium and coma are a frequent source of morbidity for ICU patients. Several factors are associated with the prognosis of mechanically ventilated (MV) cancer patients, but no studies evaluated delirium and coma (acute brain dysfunction). The present study evaluated the frequency and impact of acute brain dysfunction on mortality.

**Methods:**

The study was performed at National Cancer Institute, Rio de Janeiro, Brazil. We prospectively enrolled patients ventilated >48 h with a diagnosis of cancer. Acute brain dysfunction was assessed during the first 14 days of ICU using RASS/CAM-ICU. Patients were followed until hospital discharge. Univariate and multivariable analysis were performed to evaluate factors associated with hospital mortality.

**Results:**

170 patients were included. 73% had solid tumors, age 65 [53–72 (median, IQR 25%–75%)] years. SAPS II score was 54[46–63] points and SOFA score was (7 [6]–[9]) points. Median duration of MV was 13 (6–21) days and ICU stay was 14 (7.5–22) days. ICU mortality was 54% and hospital mortality was 66%. Acute brain dysfunction was diagnosed in 161 patients (95%). Survivors had more delirium/coma-free days [4(1,5–6) vs 1(0–2), p<0.001]. In multivariable analysis the number of days of delirium/coma-free days were associated with better outcomes as they were independent predictors of lower hospital mortality [0.771 (0.681 to 0.873), p<0.001].

**Conclusions:**

Acute brain dysfunction in MV cancer patients is frequent and independently associated with increased hospital mortality. Future studies should investigate means of preventing or mitigating acute brain dysfunction as they may have a significant impact on clinical outcomes.

## Introduction

Delirium is a common type of acute brain dysfunction in patients admitted to the intensive care unit (ICU) [1], [2]. To date, several studies have demonstrated that delirium is associated with increased risk of mortality as well as increased hospital length of stay (LOS) and costs [1]–[3]. In addition, when high-risk populations are considered, such as the elderly and mechanically ventilated, delirium may occur in up to 80% of ICU patients [2]. The impact of delirium on relevant clinical outcomes is not restricted to the hospital setting as delirium is also an independent predictor of six-month mortality and long-term cognitive impairment [2], [4], [5]. However, most epidemiological data derives from general ICU patients and critically ill cancer patients have not been thoroughly evaluated. Cancer patients may present high risk for acute brain dysfunction as it is associated with several factors such as high burden of comorbidities, chronic exposure to opioids and sedatives, acute and chronic systemic inflammation among others. Currently up to 20% of all ICU patients have a diagnosis of cancer [6], [7] and while predictors of in-hospital mortality and clinical outcomes are well described for this population [6], [8]–[10] to the best of our knowledge none of the studies investigated the occurrence and impact of delirium and acute brain dysfunction in a systematic way. The aim of the present study was to evaluate the frequency of acute brain dysfunction and its impact on outcomes of mechanically ventilated cancer patients.

## Patients and Methods

### Design and setting

The present study is a prospective cohort study performed in the ICU of Instituto Nacional de Câncer (INCA), Rio de Janeiro, Brazil. The ICU is a fifteen-bed medical-surgical unit specialized in the care of patients with cancer [8], with the exception of bone marrow transplant patients.

Briefly, during the study period (February 2010 to February 2012), every adult cancer patient (≥18 yrs) that required ICU admission was consecutively evaluated. Patients in complete remission >5 yrs, those ventilated for more than 24 h prior to ICU admission, patients ventilated for less than 48 h in the ICU and readmissions were not considered. Legal blindness and deafness and the inability to speak Portuguese as well as moribund patients (expected to die <24 h) were also excluded. The main outcome of interest was hospital mortality.

### Definitions, Selection of Participants and Data Collection

Demographic, clinical and laboratory data were collected using standardized case report forms and included main diagnosis for ICU admission, the Simplified Acute Physiology Score (SAPS) II [11] the Sequential Organ Failure Assessment (SOFA) score [12], comorbidities, and cancer- and treatment-related data. Level of arousal was measured using the RASS score [13] rates a patient's level of agitation/sedation on a 10-point scale ranging from −5 (unarousable, not responsive to voice or physical stimulation) to +4 (combative). Coma was defined as a RASS score of minus 4 (responsive only to physical stimulus) or minus 5 (unresponsive to physical stimulus) of any cause as previously defined [14]. Delirium was diagnosed with the CAM-ICU [15]. The CAM-ICU was developed for use in critically ill, intubated patients and is a validated delirium detection tool with high sensitivity and specificity and high inter-rater reliability [16] that was validated in Portuguese by our group [17]. The CAM-ICU assesses four features of delirium: (1) acute onset or fluctuating course, (2) inattention, (3) disorganized thinking, and (4) altered level of consciousness. To be considered CAM-ICU positive, the subject must display features 1 and 2, and either 3 or 4. The CAM-ICU was applied every morning by two trained investigators (I.C.A and V.C.S-D) to every eligible patient during the first 14 days of ICU stay. The ICU and hospital mortality rates from any cause were also assessed.

This study was supported by institutional funds and did not interfere with clinical decisions related with patient care. The Ethics Committee of the Instituto Nacional de Câncer in Rio de Janeiro approved the study (Number 144/2009) and the need for informed consent was waived.

### Data processing and Statistical Analysis

Data entry was performed by the investigators (I.C.A, V.C.S-D) and consistency was assessed with a rechecking procedure of a random sample of patients. Data were screened in detail by two investigators (J.I.F.S., I.C.T) for missing information, implausible and outlying values.

Standard descriptive statistics were used. Continuous variables were reported as median [25%–75% interquartile range (IQR)]. Univariate analysis was used to identify factors associated with hospital mortality. Two-tailed *P*-values <0.05 were considered statistically significant. Univariate and multivariable logistic regression were used to identify factors associated with hospital mortality. Variables yielding *P*-values below 0.2 by univariate analysis were entered into a forward multivariable logistic regression analysis. Clinically relevant variables such as: sepsis, use of sedatives, chemotherapy, cancer status, age and co-morbidities were forced into the model. Multivariable analysis results were summarized by estimating odds ratios (OR) and respective 95% confidence intervals (CI). Possible interactions were tested. The area under the receiver-operating characteristic curve was used to assess the models' discrimination. The SPSS 13.0 software package (Chicago, Illinois, USA) and Prism 3.0 (Graphpad, USA) were used for statistical analysis.

## Results

### Characteristics of the study population

**Figure 1:**
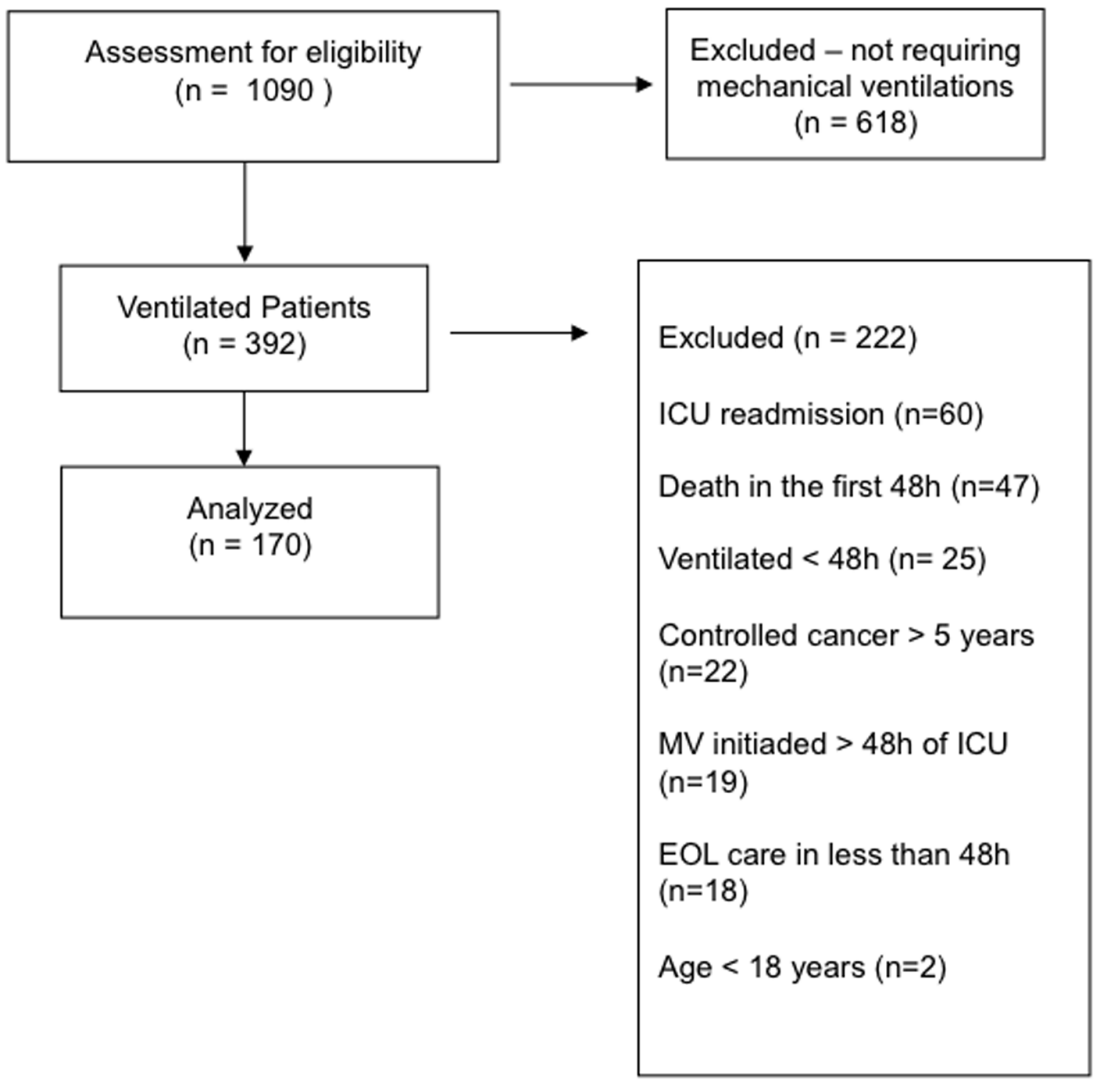
Study Flow Diagram.

After the initial screening of 1090 consecutive ICU admissions, a total of 170 patients that fulfilled inclusion criteria were enrolled in the study (Figure 1). The main characteristics including cancer-related variables of the study population are depicted in Table 1. Overall, ICU and hospital mortality were 54.7% and 66.4%%, respectively. One hundred and thirteen patients (66.4%) were admitted to the ICU due to a medical condition while emergency and elective surgery represented 25.2% and 8.2% of cases, respectively. At ICU admission, sepsis was the most frequent diagnosis (n = 108, 63.5%).

**Table 1:** Demographic and clinical variables of patients according to the presence of acute brain dysfunction.

Variables	All Patients (n = 170)	Acute Brain Dysfunction (n = 161)	No acute brain dysfunction (n = 9)	*P*-value*
**Age (years)**	63(53–72)	62(53–72)	64(50–68)	0.78
**Male gender, n (%)**	100(58.8%)	93(57.7%)	7(77.7%)	0.36
***Performance status*** ** (3–4), n (%)**	34(20%)	33(20.4%)	1(11.1%)	0.68
**Cancer status (recent diagnosis/relapse/progression), n (%)**	161(94.7%)	152(94.4%)	9(100%)	0.99
**Solid tumor, n (%)**	125(73.5%)	118(73.2%)	7(77.7%)	0.99
**Tumor extension (locally advanced/distant metastasis), n (%)**	78(45.8%)	75(46.5%)	3(33.3%)	0.64
**SAPS II score (points)**	54(46–63)	54(45–63)	57(50–60)	0.76
**Charlson comorbidity index (points)**	2(2–3)	2(2–3)	3(2–4.5)	0.50
**SOFA score (points)**	7(6–9)	7(6–9)	6(4.5–7)	0.07
**Type of admission**				
**Medical n (%)**	113(66.4%)	107(66.4%)	6(66.6%)	0.99
**Main reasons for ICU admission**				
**Sepsis, n (%)**	108(63.5%)	105(65.2%)	3(33.3%)	0.08
**Respiratory failure (excluding sepsis), n (%)**	27(15.8%)	22(13.6%)	5(55.5%)	0.006
**PaO2/FiO2 (points)**	270(200–380)	270(200–380)	270(140–390)	0.60
**Sedatives, n (%)**	168(98.8%)	161(100%)	7(77.7%)	0.002
**MV LOS (days)**	13(6–21)	13(6.5–20)	15(5–28)	0.99
**ICU LOS (days)**	14(7.5–22)	14(7–22)	13(10–20)	0.94
**Hospital LOS (days)**	26(14–39)	26(13–39)	36(21–49)	0.13
**ICU mortality, n (%)**	93(54.7%)	90(55.9%)	3(33.3%)	0.34
**Hospital mortality, n(%)**	113(66.4%)	110(68.3%)	3(33.3%)	0.06
**End of life care, n (%)**	30(17.6%)	27(16.7%)	3(33.3%)	0.25

* For comparisons among patients with and without the diagnosis of acute brain dysfunction.

SAPS II - Simplified Acute Physiology Score II; SOFA - Sequential Organ Failure Assessment; ICU - intensive care unit; LOS –length of stay; Performance is status is defined according to the Eastern Cooperative Oncology Group (ECOG) scale.

Results expressed as median (25%–75% interquartile range) and number (%).

### Diagnosis of acute brain dysfunction: Associated Characteristics and Outcomes

After excluding patients deeply sedated and unarousable with RASS deeper than −3 during the entire study period, delirium was evaluated with the CAM-ICU in 126 patients (74% of the entire eligible patient population). Daily interruption of sedation [18] was a part of routine ICU care and performed according to local protocol based on Kress et al [18].

Overall, delirium was diagnosed by the CAM-ICU in 92.8% of patients (n = 117/126) of the included arousable patients. Detailed comparisons between patients with and without a diagnosis of acute brain dysfunction (ABD) are also depicted on table 1.

Regarding hospital mortality, a comparison was performed between survivors and non-survivors (including the whole cohort). As expected, survivors presented lower severity of illness as expressed by the SAPS II scores (50 [43–60] vs 56(47–63), p = 0.011). Additionally, ventilator free-days and delirium-coma free days were higher in survivors. The results regarding the comparison of other variables are shown in Table 2.

**Table 2:** Comparison of Survivors and non-survivors.

Variables	Survivors (n = 57)	No Survivors (n = 113)	*P*-value
**Age (years)**	64(53–70.5)	62(53–73)	0.53
**Male gender, n (%)**	31(54.3%)	69(61%)	0.41
**Performance status (3–4)**	10(17.5%)	24(21.2%)	0.68
**Cancer status (recent diagnosis/relapse/progression), n (%)**	55(96.4%)	106(93.8%)	0.71
**Solid tumor, n (%)**	45(78.9%)	80(70.7%)	0.27
**Tumor extension (locally advanced/distant metastasis), n (%)**	30(52.6%)	48(42.4%)	0.25
**SAPS II score (points)**	50(43–60)	56(47–63)	0.0112
**Charlson comorbidity index (points)**	2(2–3)	2(2–3)	0.54
**SOFA score (points)**	7(5.5–9)	7(6–9)	0.60
**Type of admission - Medical, n (%)**	32(56.1%)	81(71.6%)	0.05
**Sepsis, n (%)**	37(64.9%)	71(62.8%)	0.86
**P/F score (points)**	280(190–380)	270(200–384)	0.85
**Sedatives, n (%)**	56 (98.2%)	112 (99.1%)	0.99
**Delirium/Coma**	51(89.4%)	110(97.3%)	0.06
**Delirium/coma-free days**	4(1,5–6)	1(0–2)	<0.0001
**MV LOS (days)**	9(6.5–18)	14(6–22)	0.29
**Ventilator free days (days)**	3(1–5.5)	0(0-0)	<0.0001
**ICU LOS (days)**	14.5(10–20.5)	13(6–23)	0.33
**Hospital LOS (days)**	26(25.5–53)	21(10–33)	<0.0001

Variables selected in the univariate analysis (those with p-values<0.2 and others with clinical interest regardless of p-value such as: age, charlson index, cancer type and status) were entered in multivariable analysis. In addition to the SAPSII, only acute brain dysfunction as well as delirium/coma free-days were selected in the final models and independently associated with hospital mortality (Table 3). As there was potential colinearity between the presence of acute brain dysfunction and coma-delirium free-days two models were fitted containing either the acute brain dysfunction or delirium-coma free-days. In multivariable analysis, acute brain dysfunction (OR = 5.00 [95% CI, 1,15–21.68], p = 0.03) and delirium-coma free-days (0.771 [0.681 to 0.873], p<0.001) were associated with increased hospital mortality.

**Table 3:** Multivariable analyses of factors associated with increased hospital mortality.

Variables	Coefficient	Odds-Ratio (95% CI)	*P*-value
*Model containing the Delirium/Coma*			
Delirium/Coma	1.610	5.00 (1,15–21.68)	0.03
SAPSII Score (points)	0.029	1.03 (1,002–1.059)	0.03
Surgical admission	−0.659	0,52(0.259 to 1.031)	0.06
Constant	−2.155		
*Model containing the Delirium/Coma Free Days*			
SAPSII Score (points)	0.032	1.032 (1.003 to 1.063)	0.028
Coma-Delirium Free Days	1.21	0.771 (0.681 to 0.873)	<0.001
Constant	−0.325		

Model containing the Delirium/Coma: Area under receiver operating characteristic curve = 0.67 (95% CI, 0.59 to 0.74).

Model containing the Delirium/Coma- Free Days: Area under receiver operating characteristic curve = 0.75 (95% CI, 0.68–0.81).

SAPSII - Simplified Acute Physiology Score II; CI – confidence interval.

**Figure 2:**
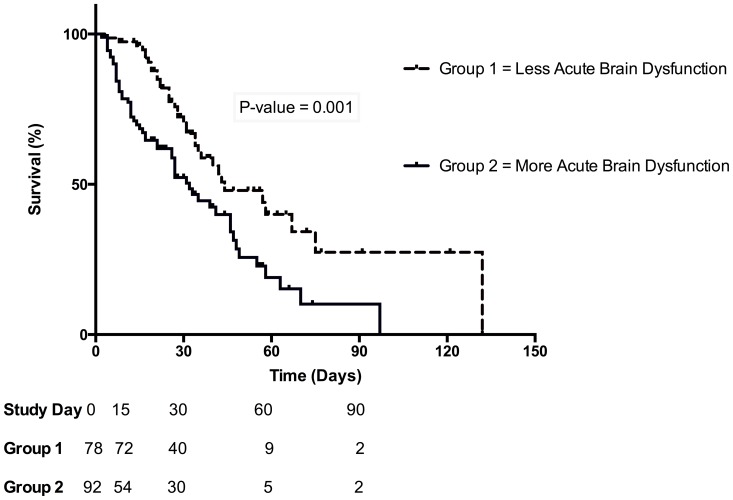
Kaplan–Meier analysis depicting the impact of delirium and coma on hospital mortality. Group 1- Less acute brain dysfunction represents patients with delirium/coma free-days >1 day. Group 2- More acute brain dysfunction represents patients with delirium/coma free-days ≤1.

**Figure 3:**
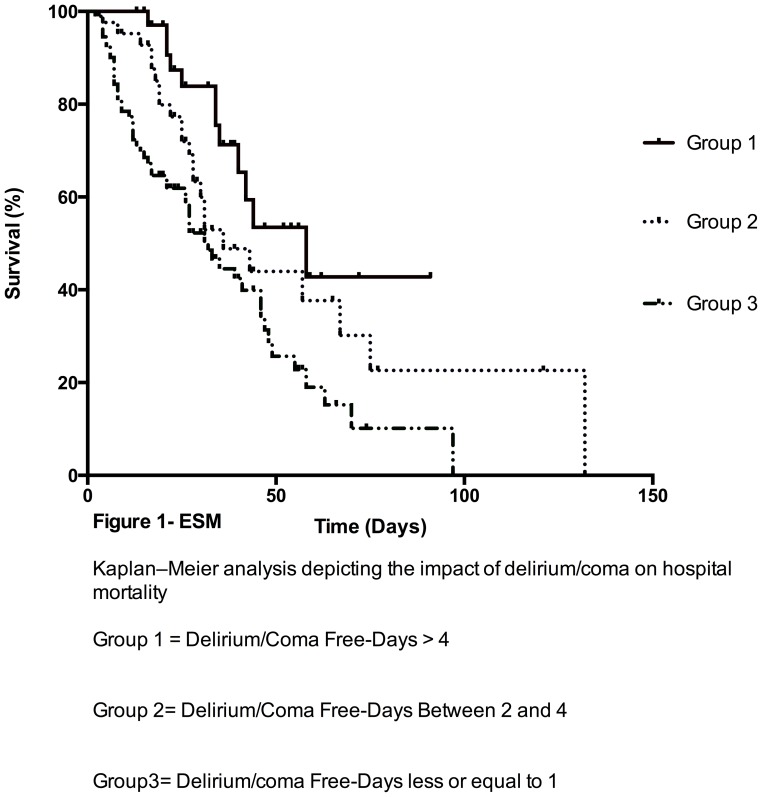
Kaplan–Meier analysis depicting the impact of delirium/coma on hospital mortality.

We also analyzed mortality of two groups stratified by the median duration of (median = 1) of delirium/coma free-days and observed higher cumulative mortality (84.8 vs 46.1%, p = 0.001) in patients that presented more acute brain dysfunction (Figure 2). Data on the mortality stratified by 3 categories of duration of delirium/coma free days is also provided in Figure 3.

Understanding the evidence of a spectrum that encompasses delirium, coma plus delirium and coma, we analyzed separately those patients that were comatose though all the study period. As expected when the 44 patients with RASS deeper than −3 for the whole study period were compared to the remaining 126 patiens we observed that they had higher SOFA scores ( 8[6–10] vs 7[5–8], p = 0.07), less ventilator-free days ( 0[0-0] vs 1[0–1], p<0.01), increased ICU (93.1% vs 41.2%, p<0.0001) and hospital mortality (95.4% vs 56.3%, p<0.0001) as compared to arousable patients regardless of diagnosis of delirium.

## Discussion

In the present study, we evaluated a prospective cohort of mechanically ventilated patients with cancer patients and observed that the frequency of acute brain dysfunction is considerably high. Moreover, acute brain dysfunction is a major predictor of mortality for this population.

In the past decade, several studies increased the knowledge of factors associated with hospital mortality for critically ill patients with cancer [8], [10], [19], [20]. These studies demonstrated that the severity of acute illness and organ dysfunctions [10] as well as patients' comorbid conditions and performance status were important determinants of short-term outcomes. The knowledge of these factors have been considered important to aid the bedside clinician to avoid forgoing intensive care for patients with a chance of survival and to improve resource allocation [10], [21], [22].

Global mortality rates observed in our population are exceedingly high, however are comparable to studies enrolling cancer patients with severe sepsis or those necessitating ventilatory support [6], [9]. Although one recognizes the importance of knowing the classic predictors of mortality in critically ill cancer patients, it should be stressed that none of them are modifiable giving clinicians little room for interventions other than a well structured ICU triage procedure and discussions on EOL care. In this sense the information that acute brain dysfunction is frequent and associated with poor outcomes in this population may be useful to test the effectiveness of interventions and help improve the current mortality rates. Several studies have demonstrated that different pharmacologic and non-pharmacologic interventions may reduce the incidence of acute brain dysfunction in mechanically ventilated patients in general ICUs [14], [23]–[25]

Several factors may help explain why patients with cancer present a high frequency of acute brain dysfunction such as chronic pain and opiod use, chronic sustained systemic inflammation, older age, high burden of comorbidities, use of steroids and terminal illness [26], [27]. Studies evaluating non-ICU cancer patients requiring hospitalization have demonstrated that delirium occurs in up to 42% of patients [28], [29]. In a recent study that evaluated patients submitted to esophageal resection delirium occurred in 50% of the patients in the post-operative period and associated with increased duration of mechanical ventilation and hospital stay [30]. However, data on critically ill cancer patients, especially the mechanically ventilated, are scarce.

The present study has some limitations. First it was a single-center study performed at a specialized center, however the patients' characteristics did not differ significantly from those in multicenter studies [6], [7]. Also, the sample size although calculated based on the mean prevalence of delirium in mechanically ventilated in contemporaneous studies [14], [23] ended up being limited and precluding subgroup analysis such as sepsis, sedative use and other relevant characteristics and risk factors and due to the unexpectedly high rate of acute brain dysfunction. Therefore it was underpowered for comparison among groups such as delirium and no-delirium. Additionally, delirium was evaluated only once a day and as it is a fluctuating syndrome some diagnoses may have been missed. However, due to the already elevated rates of acute brain dysfunction observed in our cohort we believe this impact would not be as important as if we were in a setting with lower overall rates. In addition, the fact of being performed in a specialized unit did not allow a “control group” with non-cancer patients. A study by Neufeld et al have demonstrated hat in non-critically ill hospitalized cancer patients, the CAM-ICU and ICDSC intensive care delirium screening tools are not adequately sensitive for use in routine clinical practice, although this could be a potential issue, the fact that our rates of acute brain dysfunction were very high diminishes the potential impact of such finding [31].

Also delirium subtype (a relevant clinical feature) was not evaluated. Also, we did not evaluate adherence to process of care measures that could impact in the frequency of delirium, although the unit has implemented sedation protocols as standard of care [32]. Aspects related to the cumulative dose and sedation depth over time were not registered. Therefore it was not possible to perform a comparison of patients stratified by the presence or absence of modifiable risk factors of delirium. And finally, no long-term follow-up was performed and therefore from present data we cannot draw conclusions on the impact of ABD on long-term cognitive function and quality of life of these patients. Importantly, as a cohort study, we demonstrated the association of acute brain dysfunction (a potentially modifiable predictor of outcome) and hospital mortality in mechanically ventilated cancer patients. However, a clinical trial is required to clearly demonstrate causal relation between interventions that reduce the frequency and duration of acute brain dysfunction will improve hospital survival in critically ill cancer patients.

## Conclusions

In conclusion, acute brain dysfunction is present in most mechanically ventilated cancer patients and is independently associated with mortality. Strategies aiming at the reduction of the frequency, severity and duration of this condition should be implemented in this population and tested in a population of critically ill patients with cancer.
